# Spatial prediction of animal plague risk in Qinghai Province, China using MaxEnt modeling: implications for targeted control

**DOI:** 10.3389/fvets.2026.1737541

**Published:** 2026-04-28

**Authors:** Yongshun Wang, Xinyuan Tang, Zhijie Yang, Zhoupeng Ren, Jianguo Yang, Aiping Zhang, Yecheng Yuan, Kuizhang Zhou, Ke Jiang, Angqian Duojie, Youwen Wei, Peisong You, Yongqiang Geng, Shoubiao Xu, Qingwen Zhang

**Affiliations:** 1 Qinghai Institute for Endemic Disease Prevention and Control, Xining, China; 2 State Key Laboratory of Resources and Environmental Information System, Institute of Geographic Sciences and Natural Resources Research, Chinese Academy of Sciences, Beijing, China; 3 School of Civil Engineering and Geomatics, Shandong University of Technology, Zibo, China; 4 College of Resources and Environment, University of Chinese Academy of Sciences, Beijing, China

**Keywords:** exposed population estimation, MaxEnt, plague, Qinghai province, transmission risk mapping

## Abstract

**Background:**

Plague, as a highly infectious and fatal zoonotic disease, has shown a resurgence in multiple regions worldwide in recent years. Qinghai Province, a significant natural plague focus of the plateau type in China, has experienced periodic outbreaks. However, existing research has primarily focused on the spatial prediction of marmot-suitable habitats, with relatively few studies targeting the spatial prediction of animal plague risk zones.

**Methods:**

Data on animal plague occurrence sites in Qinghai Province from 1956 to 2022 were collected. Terrain features, bioclimatic variables, land use types, and human disturbance variables related to plague spatial distribution were extracted. A maximum entropy (MaxEnt) model was used to construct a spatial prediction model for animal plague risk zones, and The model’s performance was evaluated using the average Area Under the Curve (AUC) and True Skill Statistic (TSS) values on the testing datasets. Based on the model results, the animal plague risk zones were delineated, and the area size and population residing within predicted risk zones were calculated.

**Results:**

The Maxent model achieved average AUC and TSS values of 0.827 and 0.5, respectively, on the testing datasets. The predicted animal plague risk zones in Qinghai Province covers 234,000 km^2^ (approximately 32.4% of the total area of Qinghai), with a population of 3.902 million residing within predicted risk zones (65.8% of the province’s total population).

**Conclusion:**

This study utilized the MaxEnt model combined with GIS spatial analysis technology to predict the spatial distribution of animal plague risk zones in Qinghai Province and estimate the area size and population residing within predicted risk zones. The findings can provide a scientific basis and decision-making support for targeted plague prevention and control in Qinghai.

## Background

Plague is a fatal infectious disease caused by *Yersinia pestis*, classified as a zoonosis characterized by acute onset, rapid transmission, high fatality rate, and strong infectivity (1). According to the Law of the People’s Republic of China on the Prevention and Treatment of Infectious Diseases, plague is categorized as a Class A infectious disease (2). As a typical natural focal disease, rodents (including gerbils, Himalayan marmot (*Marmota himalayana*), and voles) serve as its primary reservoir hosts, while fleas act as vectors, facilitating pathogen transmission between animals or from animals to humans through bites (3). Since the 6th century, three major pandemics of plague have resulted in over 200 million deaths, profoundly impacting human society and historical development (4). Although modern plague prevention and control measures have effectively reduced human cases, animal plague outbreaks and sporadic human cases still occur. Factors such as climate change and land-use alterations may contribute to its resurgence (5, 6). Currently, plague incidence is rebounding in the Americas, Asia, and Africa, posing a severe threat to human society due to its high infectivity and lethality (7). Since the 21st century, global plague epidemics have exhibited distinct regional patterns (8). Epidemiological data indicate that over 95% of human cases are concentrated in Africa, with the Democratic Republic of Congo, Madagascar, and Uganda being the most severely affected (9, 10). Additionally, countries that had reported no cases for decades have experienced resurgences—for instance, Libya reported five cases in 2009 after 25 years of silence, and Kyrgyzstan confirmed one case in 2013 after a 32-year hiatus (11, 12). China, as a country with natural plague foci, continues to report sporadic human cases (13). From 2001 to 2020, the country recorded 252 human cases and 44 deaths (14). Between 2011 and 2020, Qinghai Province reported one fatal human case and 16 animal plague outbreaks (15). These surveillance data indicate that plague remains a persistent public health risk. Since animal plague outbreaks are a critical precursor to human cases, enhanced monitoring and prevention of animal plague can serve as an early warning system, thereby reducing the risk of human outbreaks (16).

Qinghai Province is a crucial natural plague focus of the plateau type in China, with the Himalayan marmot (*Marmota himalayana*) serving as the primary reservoir host. The distribution and activity of this species directly influence the epidemiological characteristics of plague in this region (17). Therefore, investigating the factors influencing the distribution of the Himalayan marmot helps to elucidate the transmission mechanisms of animal plague and provides a scientific basis for predicting plague risk zones in Qinghai Province (18). Previous studies have demonstrated that within specific regions, geographic environmental factors (including climate, vegetation, and soil) determine the spatiotemporal distribution of Himalayan marmots (19). First, elevation regulates temperature, vegetation types, and seasonal variations, determining the annual activity patterns of marmots and confining their primary distribution to alpine meadow zones at mid-to-high altitudes (20, 21). Second, topographic variables like slope and aspect effectively shape marmot habitats. For instance, sun-facing slopes with suitable temperatures and lush vegetation are preferred, while gentle slopes facilitate burrow excavation, drainage, and concealment needs (22). Ecologically, soil types indirectly influence marmot distribution by affecting plant growth and burrow stability (23). Climatic factors (e.g., mean annual temperature, precipitation, and land surface temperature) further shape their spatial distribution by regulating food availability, water resources, and hibernation conditions (24). Meanwhile, vegetation types significantly determine marmot distribution, particularly alpine meadows that provide optimal habitats and abundant food resources (25). Additionally, human disturbances such as road/railway construction, settlement expansion, and increased population density have significantly constrained marmot distribution patterns through habitat encroachment and environmental modification (26). Investigating these environmental and anthropogenic factors not only reveals spatial distribution patterns of marmots but also provides critical ecological indicators for predicting animal plague risk zones.

Qinghai Province’s vast territory and complex geographical environment make it difficult for isolated plague-positive surveillance data to comprehensively reflect the animal plague’s distribution across the entire province. Additionally, in sparsely populated and inaccessible regions, traditional field-based trapping and testing of Himalayan marmots present inevitable surveillance gaps (19). Consequently, predicting animal plague risk zones in Qinghai based solely on limited surveillance data carries significant limitations. To address this challenge, researchers have recently explored integrating ecological niche modeling with geographic information systems (GIS) (27). A predictive model was constructed to predict the spatial distribution of the Himalayan marmot by integrating its occurrence records with relevant eco-environmental variables. For example, Gao et al. took Wulan County in Qinghai Province as the study area, utilizing the MaxEnt model and ArcGIS 10.8 software to construct a predictive model for the spatial distribution of the Himalayan marmot, thereby predicting its spatial distribution (28). Similarly, Duojie et al. also employed the MaxEnt model to predict the spatial distribution of the Himalayan marmot in the Golog Tibetan Autonomous Prefecture of Qinghai Province (29). However, existing studies suffer from twofold limitations. Firstly, regarding spatial scale, their predictive scopes are mostly confined to local plague foci within Qinghai Province (such as Wulan County and Golog Tibetan Autonomous Prefecture), making province-wide coverage difficult to achieve. Secondly, concerning research content, current studies predominantly focus on the spatial prediction of suitable habitats for marmots, while relatively few studies address the spatial prediction of animal plague risk zones.

This study utilized the MaxEnt model to predict the occurrence probability of animal plague in Qinghai Province, based on historical occurrence point data of animal plague, combined with environmental variables and human activity disturbance variables. It should be emphasized that the predictive results of this model do not reflect the real-time transmission intensity of the epidemic, but rather assess the potential probability of plague occurrence among animals in the region. Based on these probability predictions, we further calculated the area of risk zones and the population size residing within these zones, thereby providing a scientific basis for precise prevention and control of animal plague and the allocation of medical resources in Qinghai Province.

## Methods

### Study area

**Figure 1:**
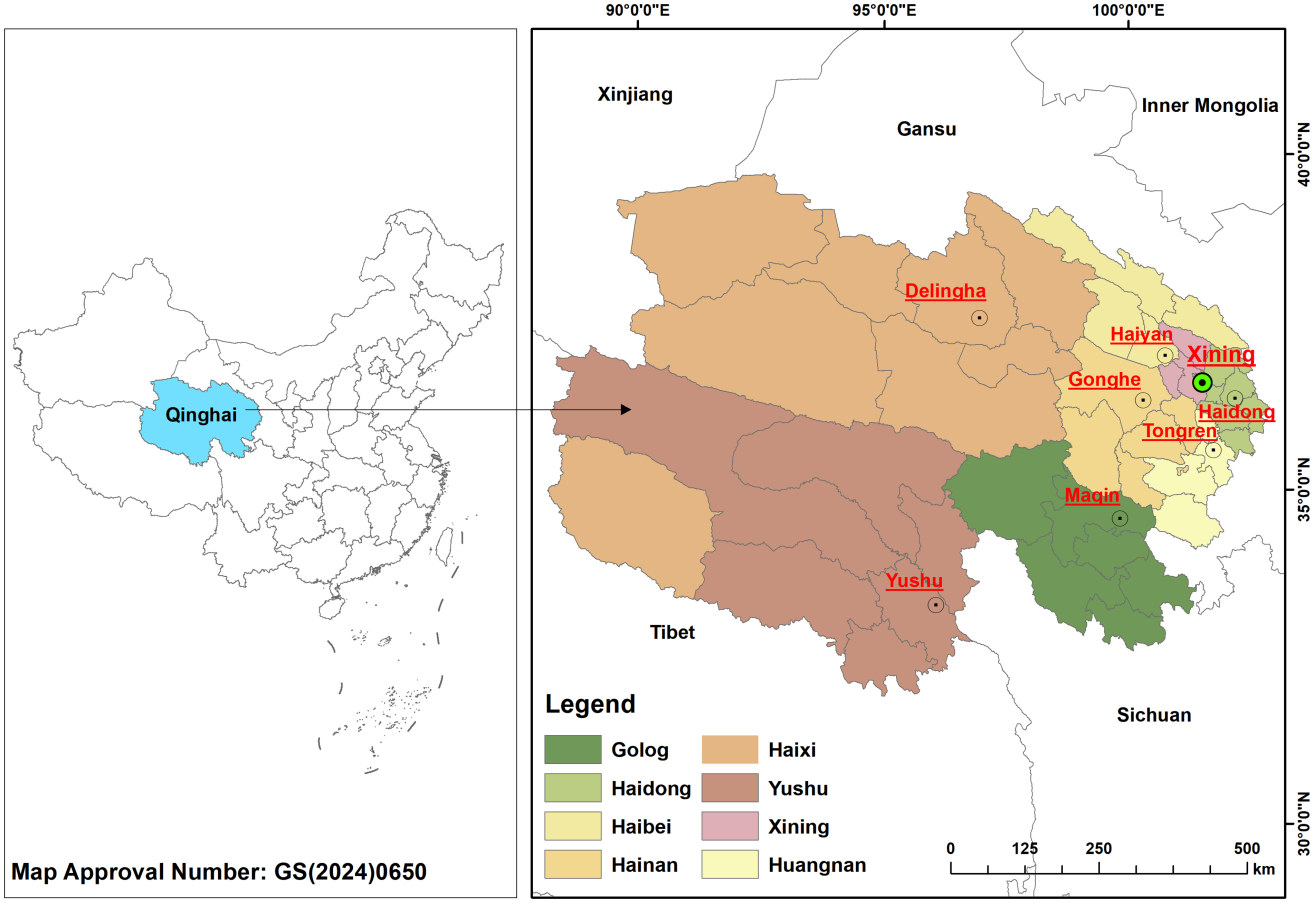
Location of Qinghai Province, China.

Qinghai Province is situated between 31°36’N ~ 39°19’N latitude and 89°35′E ~ 103°04′E longitude, comprising 2 prefecture-level cities and 6 autonomous prefectures with a permanent population of 5.93 million (as shown in Figure 1) (30). The total area spans 722,300 km^2^. The marmot plague natural foci covers approximately 200,000 km^2^, accounting for 27.74% of the province’s total area (19). The region features a typical cold continental climate, with mean annual temperatures below 9 °C and annual precipitation ranging 250–550 mm (concentrated July–September) (31). The province generally features higher elevations in the west, north, and south, and lower elevations in the east and central areas, with an average elevation exceeding 3,000 m. These arid climatic conditions and complex topographical features create ideal habitats for reservoir hosts like marmots while facilitating the persistent circulation and transmission of *Yersinia pestis* in natural plague foci (32, 33).

## Data sources

### Animal plague occurrence data

Data on animal plague occurrence sites in Qinghai Province, China, from 1956 to 2022 were provided by the Qinghai Institute for Endemic Disease Prevention and Control. In this study, an animal plague occurrence site was strictly defined according to the National Scheme of Plague Surveillance of China, referring specifically to locations within Qinghai Province where *Yersinia pestis* was successfully isolated and detected from primary host animals (such as the Himalayan marmot) or their vectors (fleas). During the surveillance of animal plague, disease control professionals conducted field surveys along designated routes within natural plague foci during the marmots’ active season. Samples were obtained by live-trapping hosts, searching for naturally dead animals, and collecting vector fleas. Subsequently, rigorous laboratory dissections and bacteriological isolation and cultivation were performed to confirm the presence of *Y. pestis*. The specific locations where the bacteria were detected were then officially recorded as animal plague occurrence sites.

**Figure 2:**
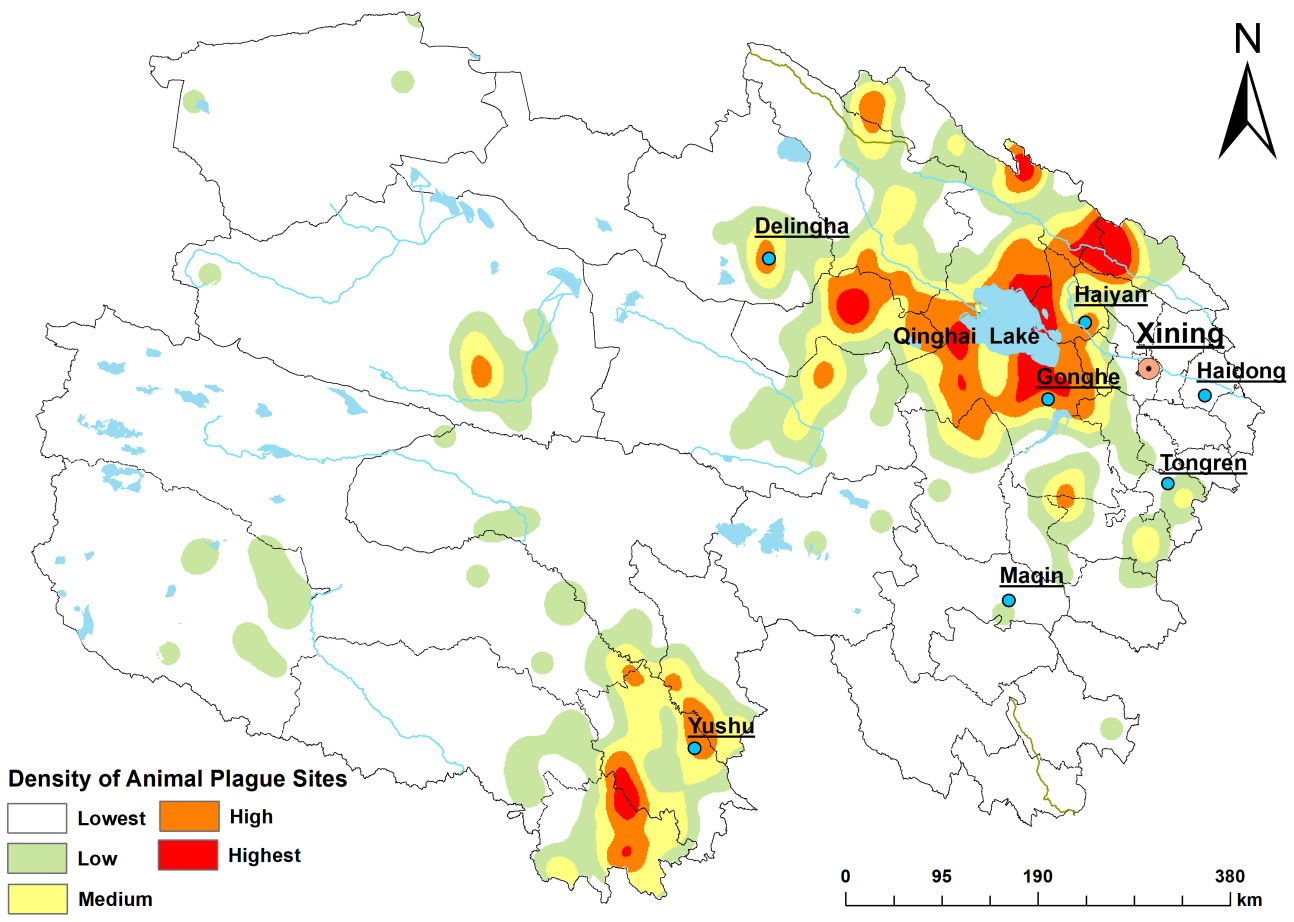
Spatial distribution heatmap of animal plague occurrence sites in Qinghai Province, China (1956–2022).

Regarding data acquisition, the original records were derived from two primary sources: (1) early historical archives, including epidemiological investigation reports, historical statistical charts, and official documents detailing confirmed plague locations; and (2) the plague surveillance database established by the Qinghai provincial disease control departments. By systematically organizing and integrating these historical archives and database records, we initially compiled a total of 836 records of animal plague occurrence sites between 1956 and 2022. To ensure the accuracy of the spatial analysis, the integrated dataset underwent rigorous data cleaning. Redundant records with spatial overlap and duplicate information were excluded, yielding a final dataset of 650 unique and valid occurrence records of animal plague. Subsequently, the text-based addresses of these valid sites were geocoded into longitude and latitude coordinates using the Amap Open Platform (API) for precise spatial positioning. Based on these coordinates, a spatial distribution heatmap of animal plague occurrence sites in Qinghai Province was generated (Figure 2).

### Climatic variables

Bioclimatic variables characterize long-term climatic conditions at a height of 2 meters above the ground, reflecting the persistent influence of temperature, precipitation, and other factors on host population dynamics. Land surface temperature, on the other hand, directly represents the thermal conditions of the ground, and the spatial annual averages of its long-term time-series data can indicate stable environmental features of a region. Therefore, we utilized the 1 km resolution monthly maximum temperature, minimum temperature, and average precipitation gridded datasets for China from 1956 to 2022, released by the National Tibetan Plateau/Third Pole Environment Data Center (34–36). These datasets were generated using the Delta spatial downscaling method over China, based on the global 0.5° climate dataset from the Climatic Research Unit (CRU) and the high-resolution global climate dataset from WorldClim. The 19 bioclimatic variables at a 1 km spatial resolution were derived from the monthly maximum and minimum temperatures and monthly precipitation data, calculated using the *biovars* function in the R package *dismo,* Details are provided in Supplementary Table S1.

This study employed the China Land and Surrounding Areas Daily 1 km All-Weather Land Surface Temperature (LST) dataset, released by the National Tibetan Plateau/Third Pole Environment Data Center (37). The dataset was reconstructed by integrating Terra/Aqua MODIS LST products, GLDAS data, and auxiliary data such as vegetation indices and surface albedo from satellite remote sensing. It leverages thermal infrared remote sensing and spatial correlations of LST components (high-frequency and low-frequency) provided by reanalysis data to produce a high-quality all-weather LST dataset. Using the Raster Calculator tool in ArcGIS 10.8, we first calculated the average daytime and nighttime LST for Qinghai Province from two different satellites. Next, we derived the daily LST averages for each satellite, then aggregated these daily averages into monthly LST averages. Finally, the monthly LST averages were further synthesized into annual LST averages.

### Geographic environmental variables

The NDVI data were sourced from the Land Use and Global Change Remote Sensing Team at the Institute of Geographic Sciences and Natural Resources Research, Chinese Academy of Sciences. Primarily based on the Google Earth Engine cloud computing platform, the data were derived from all available Landsat 5/7/8 remote sensing images for the year 2020. Through a series of preprocessing and smoothing methods, the maximum NDVI value for each pixel over the year was obtained (38). Vegetation type data for Qinghai Province were obtained from the Resource and Environment Science and Data Center, Chinese Academy of Sciences (39). These data were digitized from the scanned 1:1,000,000 vegetation map of Qinghai Province. Common vegetation types include alpine steppe, alpine desert, deciduous broadleaf forest, and others.

This study utilized the 2020 China Land Cover Remote Sensing Monitoring Dataset, with a spatial resolution of 1 km (40). The classification system adopts a two-level hierarchy, categorizing land cover types in Qinghai Province into cropland, forest, grassland, water bodies, built-up land, and unused land, based on land resources and utilization attributes. Soil type distribution data for Qinghai Province were derived from the 1:1,000,000 Soil Type Database of China (41). The predominant soil types in Qinghai are felty soils and cold calcic soils.

Digital Elevation Model (DEM) data were sourced from the Resource and Environment Science and Data Center, Chinese Academy of Sciences (42). The dataset was generated by stitching and processing the Shuttle Radar Topography Mission (SRTM) data collected by the U. S. Space Shuttle Endeavour, resulting in 30 m resolution provincial-level data. Slope and aspect data were calculated from the DEM using the slope and aspect tools in ArcGIS 10.2. Geomorphological data for Qinghai Province were obtained from the 1:4,000,000 Geomorphological Map of China, which includes 41 geomorphological types (43). Major geomorphological types encompass mountains, hills, and others.

#### Human disturbance variables

This study is based on the 1:1,000,000 public version of fundamental geographic information data (2021 edition) released by the National Catalogue Service for Geographic Information Resources. The dataset includes key features such as roads and settlements (41). First, road and settlement layers within Qinghai Province were extracted from the national tiled data. Subsequently, using spatial analysis tools in ArcGIS, Euclidean distance was calculated to generate raster images, quantifying the straight-line distance from each pixel to the nearest road or settlement, thereby assessing regional transportation accessibility. Additionally, the 2020 China 1 km population density gridded dataset, generated by the WorldPop open platform using a random forest algorithm to integrate multi-source data (e.g., LandScan nighttime light, OSM road networks, and Sentinel satellite imagery), was employed (42). The population density raster for Qinghai Province was obtained by clipping the dataset with the provincial administrative boundary using the mask tool in ArcGIS 10.2.

### Model establishment

#### Variable selection

**Figure 3:**
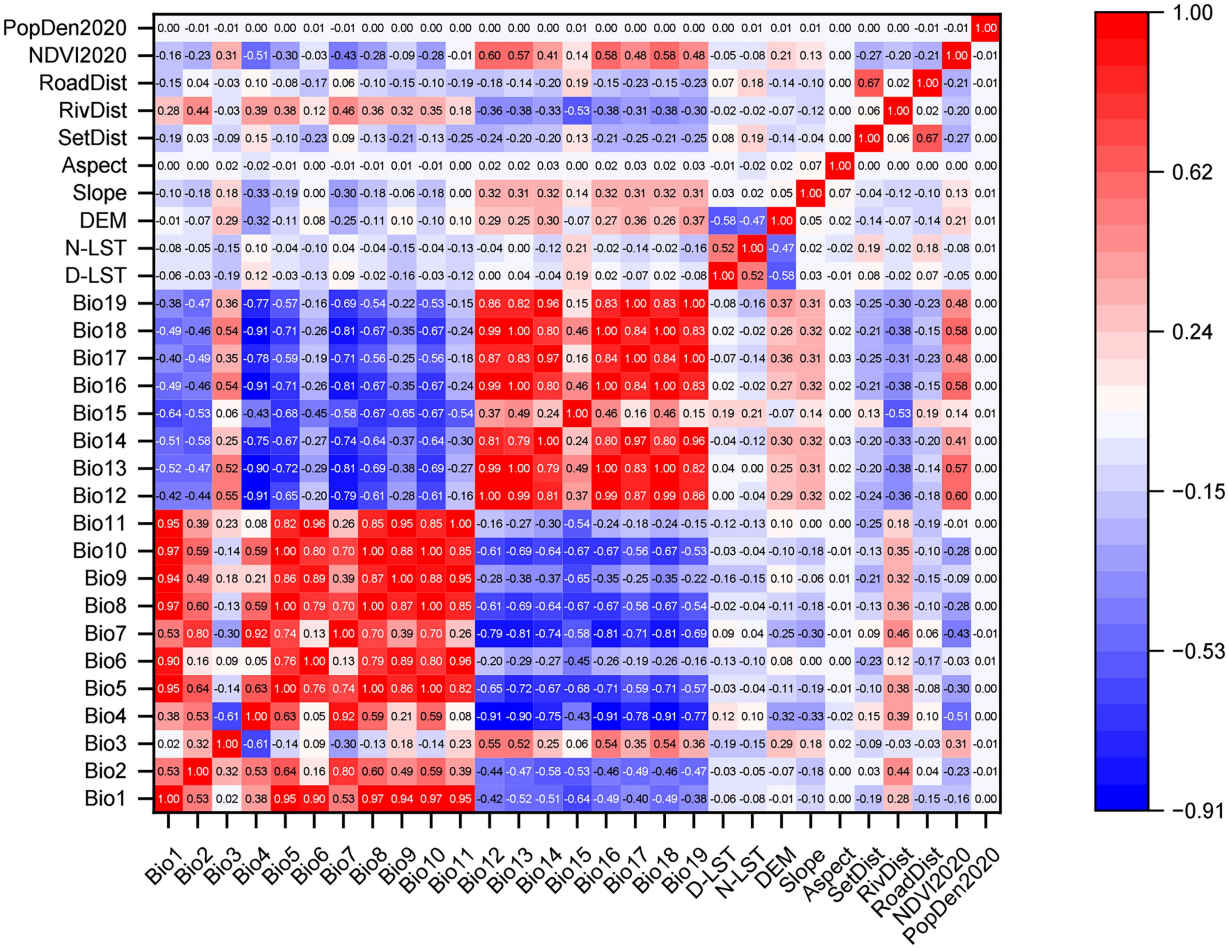
Heatmap of Pearson correlation coefficients for predictors of plague epizootic risk. Bio1–Bio19: Bioclimatic variables (detailed in Supplementary Table S1); D-LST: daytime land surface temperature; N-LST: nighttime land surface temperature; SetDist: distance to nearest settlement; RivDist: distance to nearest river; RoadDist: distance to nearest road; PopDen2020: population density (2020); NDVI2020: normalized difference vegetation index (2020).

We followed three steps to select predictive variables. First, we selected environmental variables with significant biological relevance to the survival and activity of plague host animals. Second, we employed a random forest model for univariate analysis to identify variables that could enhance the model’s predictive performance. The AUC value was used as the screening criterion; if a variable’s AUC value was below 0.5, it indicated that its predictive ability was inferior to random chance, and thus it was deemed to contribute insignificantly to model accuracy and was excluded. Based on the model’s analysis, we selected 29 continuous variables (19 bioclimatic variables, distance to the nearest residential area, distance to the nearest road, distance to the nearest river, population density, daytime land surface temperature, nighttime land surface temperature, DEM, slope, aspect, and NDVI) and 4 categorical variables (soil type, land cover type, geomorphological type, and vegetation type), all of which significantly improved the model’s predictive performance. Finally, we ensured that the selected continuous predictive variables exhibited no strong collinearity, i.e., the correlation coefficient between variables was less than 0.75. If the environmental variables in the dataset exhibited multicollinearity, it would severely compromise the reliability of the MaxEnt model’s predictions. Therefore, we used Pearson correlation coefficients to analyze the correlations among continuous variables. A heatmap revealed that some climatic variables, such as Bio1 and Bio10, had a correlation coefficient as high as 0.97, indicating significant collinearity between them, as shown in Figure 3. To eliminate multicollinearity among environmental variables, we first performed z-score normalization on the 33 variables. Subsequently, using principal component analysis (PCA) and the cumulative variance threshold method, we selected five linearly independent principal components. The specific variables represented by each principal component are detailed in Supplementary Table S2. Specifically, principal component 1 (PC1) is primarily represented by the maximum temperature of the warmest month (Bio5), mean temperature of the warmest quarter (Bio10), elevation (DEM), and precipitation of the wettest month (Bio13). Principal component 2 (PC2) is mainly represented by the mean temperature of the coldest quarter (Bio11) and the distance to residential areas (SetDist). Principal component 3 (PC3) is primarily represented by the mean diurnal range (Bio2) and isothermality (Bio3). Principal component 4 (PC4) is mainly represented by precipitation seasonality (Bio15), and principal component 5 (PC5) is primarily represented by aspect (Aspect). Finally, these five principal components, along with four categorical variables—soil type, land cover type, landform type, and vegetation type—were ultimately selected for constructing the MaxEnt model.

### Ecological niche modeling

The MaxEnt model is a machine learning model based on the principle of maximum entropy, widely used in species distribution prediction and ecological research. In this study, we employed this model to predict the probability of animal plague outbreaks by integrating known plague occurrence data from Qinghai Province and their associated environmental and human disturbance variables. First, the 650 plague occurrence points were divided into 75% training data for model training and parameter tuning, and 25% testing data for model validation and accuracy assessment. Second, response curves were created to analyze the patterns of plague transmission risk in response to changes in individual environmental variables. The jackknife method was used to quantify the contribution of each environmental factor. In terms of model output, the logistic regression output mode of the MaxEnt model generates continuous plague probability values ranging from 0 to 1. This continuous probability output facilitates subsequent plague risk zoning. To enhance the stability of model predictions, this study adopted a repeated cross-validation strategy, running the data 30 times with subsampling as the repetition type. This method constructs multiple mutually exclusive training and testing subsets, accommodating small sample sizes while mitigating spatial autocorrelation issues in plague occurrence data, thereby significantly improving the model’s generalization ability and the reliability and stability of its predictions. Finally, the median of the predicted plague probability values was selected as the final plague probability result for Qinghai Province. The median, rather than the mean, was chosen because it is more robust to extreme values (e.g., individual grid cells with probabilities close to 0 or 1) during model runs, reducing the impact of random disturbances and yielding a more stable and reliable spatial distribution of plague risk probabilities.

### Model accuracy evaluation

The Area Under the Curve (AUC), specifically the area under the Receiver Operating Characteristic (ROC) curve, is a crucial metric for evaluating the predictive performance of MaxEnt models (43). The True Skill Statistic (TSS) serves as a complementary evaluation metric to AUC, measuring the model’s comprehensive ability to correctly identify true presences and true absences at a specific threshold (e.g., the 10th percentile training presence logistic threshold) (44). In this study, the average AUC and average TSS values from testing datasets, calculated through 30 replicated cross-validations, were used to comprehensively assess model predictive performance. AUC values range from 0.5 to 1.0, with values closer to 1 indicating higher accuracy of the model in predicting the probability of plague occurrence among animals. According to widely accepted criteria in the ecological niche modeling field (43), the interpretation of AUC values is classified as follows: 0.5–0.7 indicates low accuracy, 0.7–0.9 indicates useful accuracy, and >0.9 indicates high accuracy. TSS values range from −1.0 to 1.0, with values closer to 1.0 indicating greater consistency between the model’s predictions at a specific threshold and the actual distribution of plague events among animals. Its accuracy classification criteria are as follows: TSS < 0.40 indicates poor predictive accuracy, 0.40 ≤ TSS ≤ 0.8 indicates good predictive accuracy, and TSS > 0.8 indicates excellent predictive accuracy (45).

### Plague risk zone delineation and exposed population estimation

The zoning of animal plague risk areas and area calculations based on the MaxEnt model can reveal the spatial distribution characteristics of animal plague in natural foci within Qinghai Province, providing critical guidance for developing targeted surveillance and control strategies. In this study, the median predicted plague probability map was converted into a binary classification (1 for risk zones, 0 for non-risk zones) using the 10th percentile logistic training threshold. Areas with values above this threshold were classified as plague-endemic zones, while those below were designated as non-endemic zones. This threshold method effectively balances the sensitivity and specificity of model predictions by excluding the probability values corresponding to the 10% most extreme outlier distribution points in the training data (46), making it particularly suitable for plague occurrence datasets with sampling bias.

To accurately quantify the extent of plague risk zones, this study estimated the area of plague-endemic zones within each township through spatial statistical methods based on the binary plague probability map and township administrative boundaries, and generated spatial distribution maps of plague-endemic zones by township. To estimate the exposed population within plague risk zones, this study utilized 1 km-resolution population density raster data and the binary plague probability map. Through spatial analysis methods, the number of exposed populations in plague risk zones was quantified for each township, and spatial distribution maps of exposed populations by township were generated.

## Results

### ROC curve results

**Figure 4:**
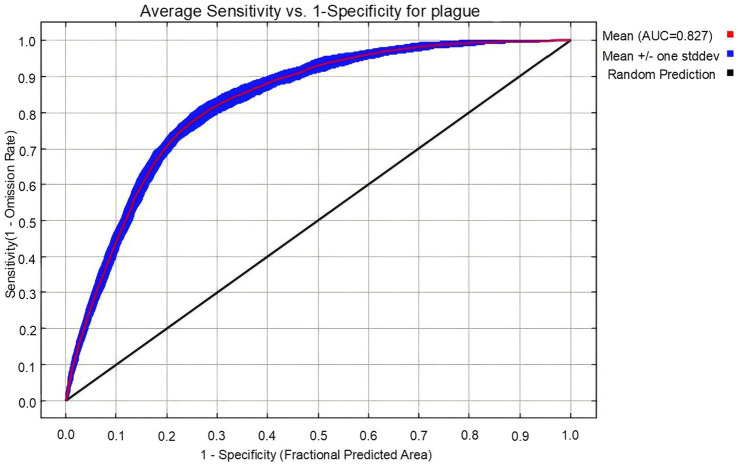
ROC curve of the MaxEnt model for predicting plague risk areas in Qinghai Province.

Based on the ROC curve of the model, the MaxEnt model achieved an average AUC value of 0.827 (±0.009) on the testing datasets and an average TSS value of 0.5, which was classified as a good level of predictive accuracy, indicating that the model can effectively predict the probability of plague prevalence, as shown in Figure 4.

### Contribution rates and response curves of variables

**Figure 5:**
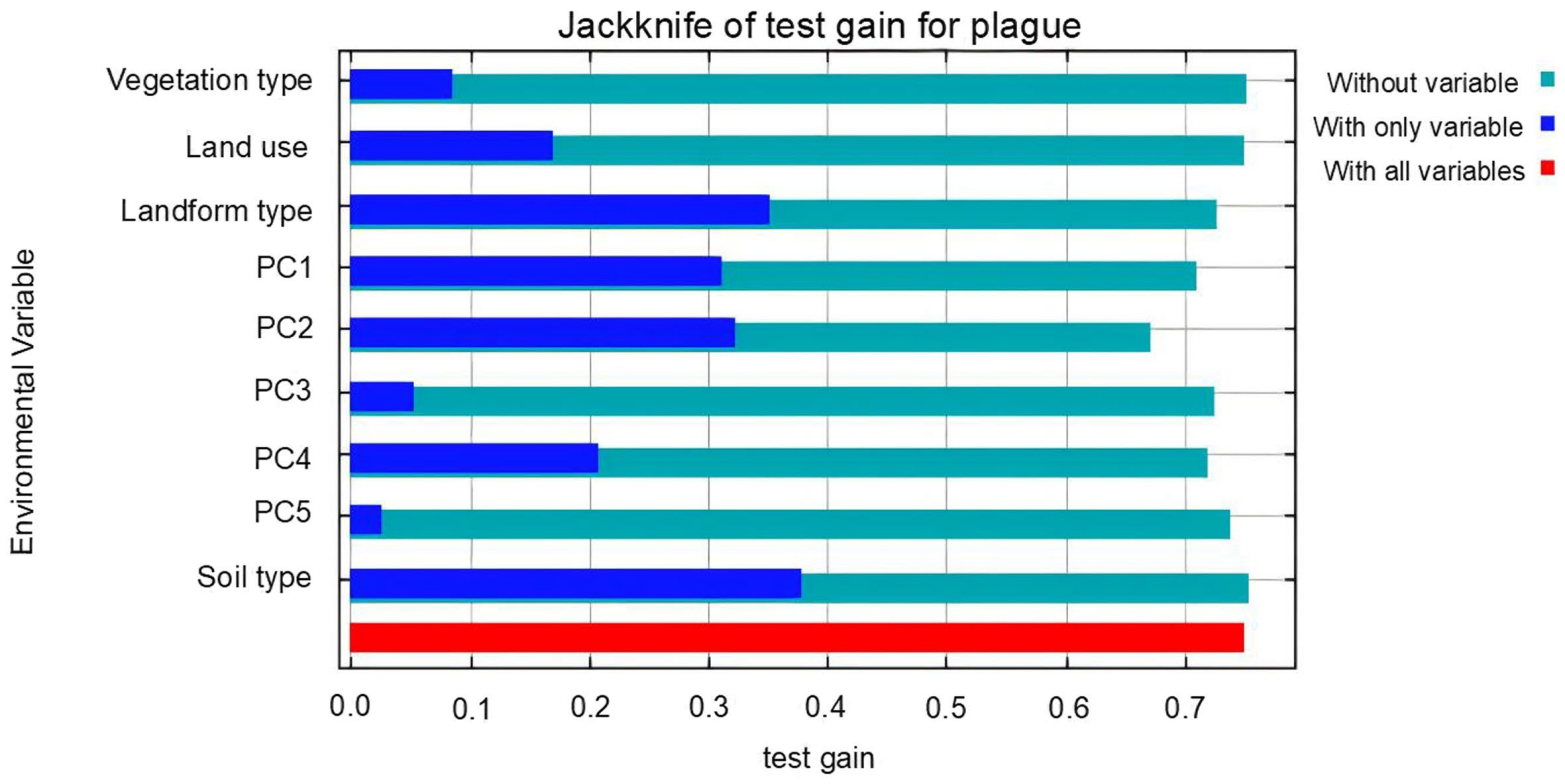
Jackknife test scores of predictor variables for plague prediction. The green bands indicate the degree of specificity of the variable, with shorter bars indicating that the variable contains unique information and is more likely to influence the distribution of the species. The blue bands indicate the variable’s effectiveness on the species’ distribution, with longer bars indicating that the variable contains more effective information. All bioclimatic variables utilized in the model are deemed essential.

The percentage contribution of each variable reflects its influence on the plague prediction model. We found that the environmental variables ranked by their contribution to the model, from highest to lowest, were: Principal Component 2 (28.6%), Principal Component 1 (18.6%), soil type (17.2%), geomorphological type (15.7%), Principal Component 4 (11.7%), land use type (4.4%), Principal Component 3 (1.8%), vegetation type (1.2%), and Principal Component 5 (0.7%). Additionally, in the variable importance jackknife plot (Figure 5), the green bars represent the specificity of each variable. Shorter bars indicate that the variable contains unique information and is more likely to influence the probability of plague occurrence. The results show that Principal Component 2 and Principal Component 1 are critical factors affecting plague occurrence, which aligns with the variable importance findings. The blue bars represent the effectiveness of variables in species distribution modeling, with longer bars indicating that the variables contain more informative content. Thus, all variables included in the model are indispensable.

**Figure 6:**
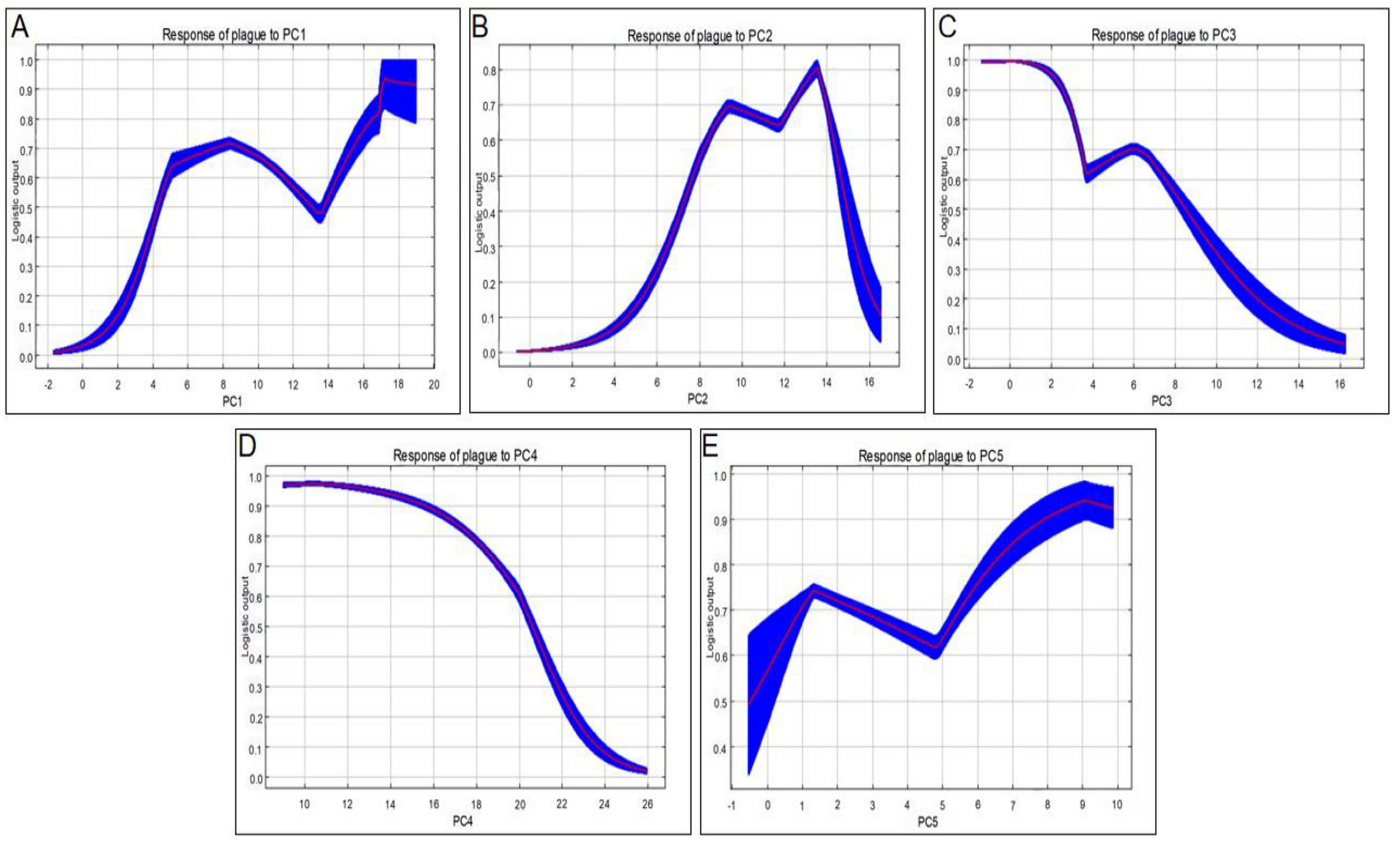
Response curves of principal components for plague epizootic risk prediction. **(A)**Principal Component 1, **(B)** Principal Component 2, **(C)** Principal Component 3, **(D)** Principal Component 4, **(E)** Principal Component 5. The curves show the mean response of the 30 replicate Maxent runs (red) and and the mean +/− one standard deviation (blue).

The variable contribution rates and response curves derived from the MaxEnt model (Figure 6) indicate that PC2, PC1, and PC4 are the primary variables driving the occurrence of animal plague in Qinghai Province. The geographic and environmental factors represented by these principal components collectively shape the spatial distribution of the disease. PC2 made the highest contribution to the model. Its response curve reveals a fluctuating trend in the probability of plague occurrence as the PC2 value increases—initially decreasing, followed by a brief rise, and ultimately declining. Interpreted alongside the PCA loading matrix (Supplementary Table S2), this pattern suggests that animal plague is more likely to occur in environments located closer to human settlements (SetDist) and characterized by higher mean temperatures during the coldest quarter (Bio11). Furthermore, PC1 emerged as another key driving variable, with the probability of plague occurrence peaking when the PC1 value falls within the 16.0–18.0 range. This indicates that the habitats most highly suitable for animal plague are characterized by higher maximum temperatures in the warmest month (Bio5) and higher mean temperatures in the warmest quarter (Bio10), coupled with lower elevations (DEM) and reduced precipitation during the wettest month (Bio13). Finally, the response curve for PC4, which primarily represents precipitation seasonality (Bio15), demonstrates a gradual decrease in occurrence probability as the component value increases. This highlights the intra-annual distribution pattern of precipitation as a critical factor influencing animal plague outbreaks. Consequently, the epidemic risk of animal plague in Qinghai Province is jointly constrained by a combination of natural climatic and topographic factors—such as the mean temperature of the coldest quarter, maximum temperature of the warmest month, elevation, and precipitation seasonality—alongside human disturbance factors, notably the proximity to residential areas.

**Figure 7:**
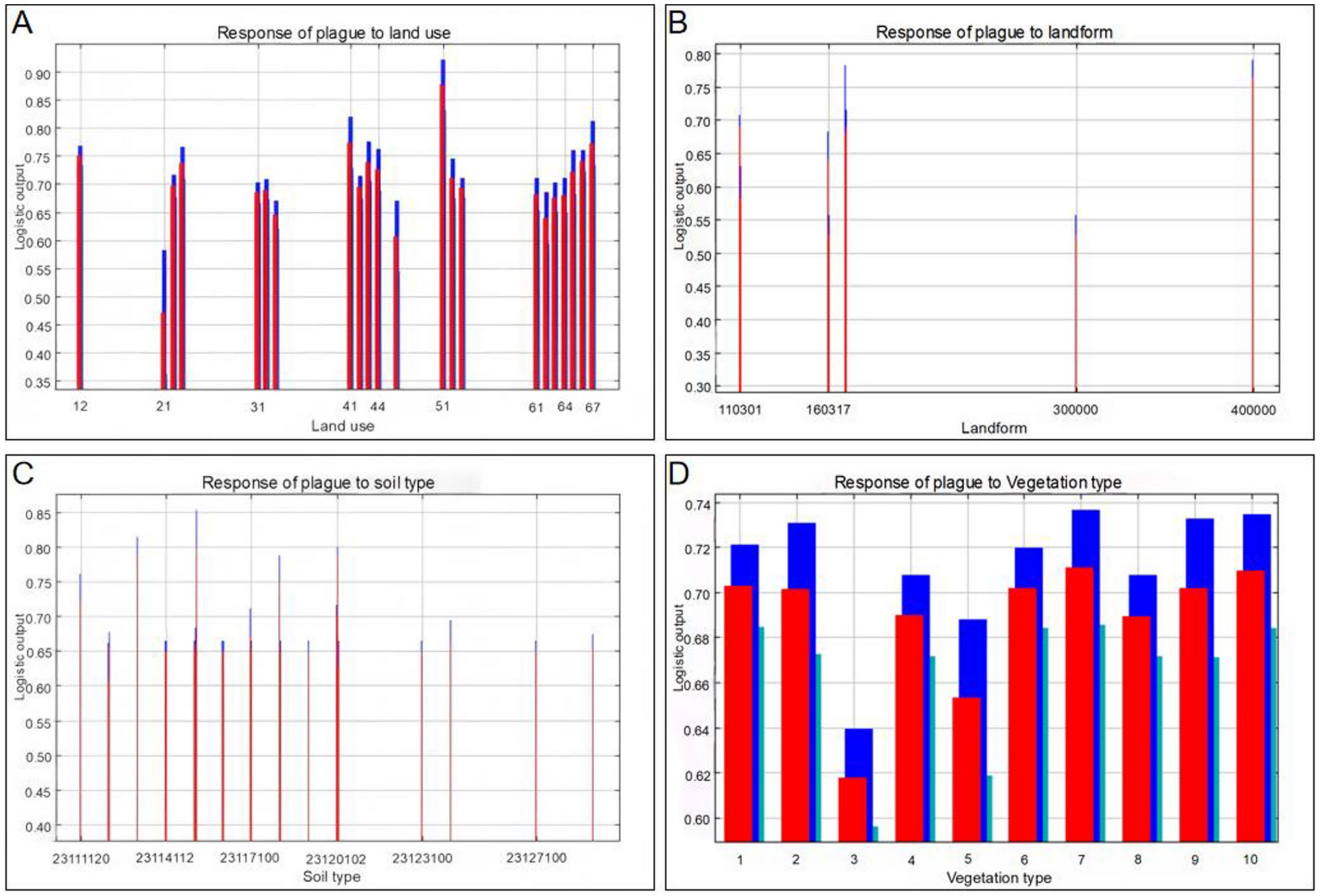
Response curves of categorical environmental variables in plague epizootic risk prediction. **(A)** Land use: 12 Dryland, 21 Forest Land, 31 High-coverage Grassland, 41 Rivers and Canals, 44 Permanent Glacier and Snow Cover, 51 Urban Land, 61 Sandy Land, 64 Marshland, 67 Unused Land. **(B)** Landform type: 110301 Moderate-relief High Mountains, 160,317 Low-relief High Mountains, 300,000 High-relief High Mountains, 400,000 High-relief High Mountains. **(C)** Soil type: 23111120 Cinnamon Soil, 23,114,112 Gray-brown Desert Soil, 23,117,100 Gleyed Soil, 23,120,102 Mattic Cryic Soil, 23,123,100 Meadow Swamp Soil, 23,127,100 Saline Soil. **(D)** Vegetation type: 1 Deciduous Broadleaf Forest, 2 Evergreen Coniferous Forest, 3 Deciduous Broadleaf Scrub, 4 Evergreen Coniferous Scrub, 5 Steppe, 6 Shrub and Small Tree Desert, 7 Semi-Shrub Desert, 8 Alpine Scrub, 9 Alpine Meadow, 10 Alpine Steppe.

Among land use types, agricultural landscapes such as cropland and grassland exhibited higher occurrence probabilities, whereas the response in areas covered by natural vegetation was relatively lower. From the perspective of topographic factors, the risk of plague prevalence in medium-altitude regions was significantly higher than that in extreme terrain. Furthermore, the response curves for soil and vegetation types further confirmed that ecological conditions such as sandy soils and alpine meadows are more conducive to the prevalence and transmission of plague, as shown in Figure 7.

### Plague risk zone and exposed population estimation results

**Figure 8:**
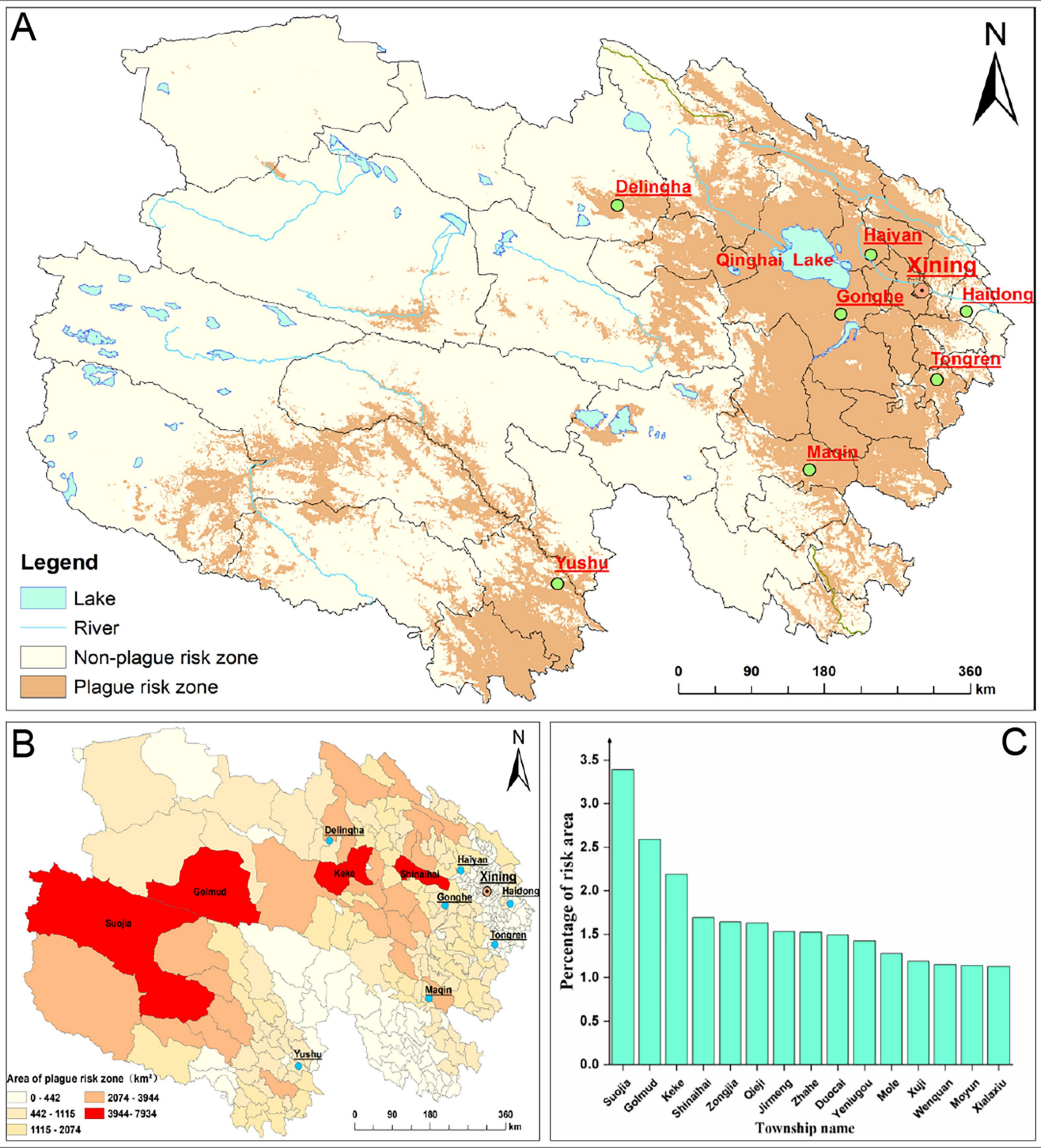
Spatial distribution and risk area proportion of plague risk zones in townships of Qinghai Province. **(A)** Spatial distribution of plague risk zones in Qinghai; **(B)** spatial distribution of plague risk zone areas in townships; **(C)** area proportion of plague risk zones in the top 15 high-risk townships of Qinghai Province.

**Figure 9:**
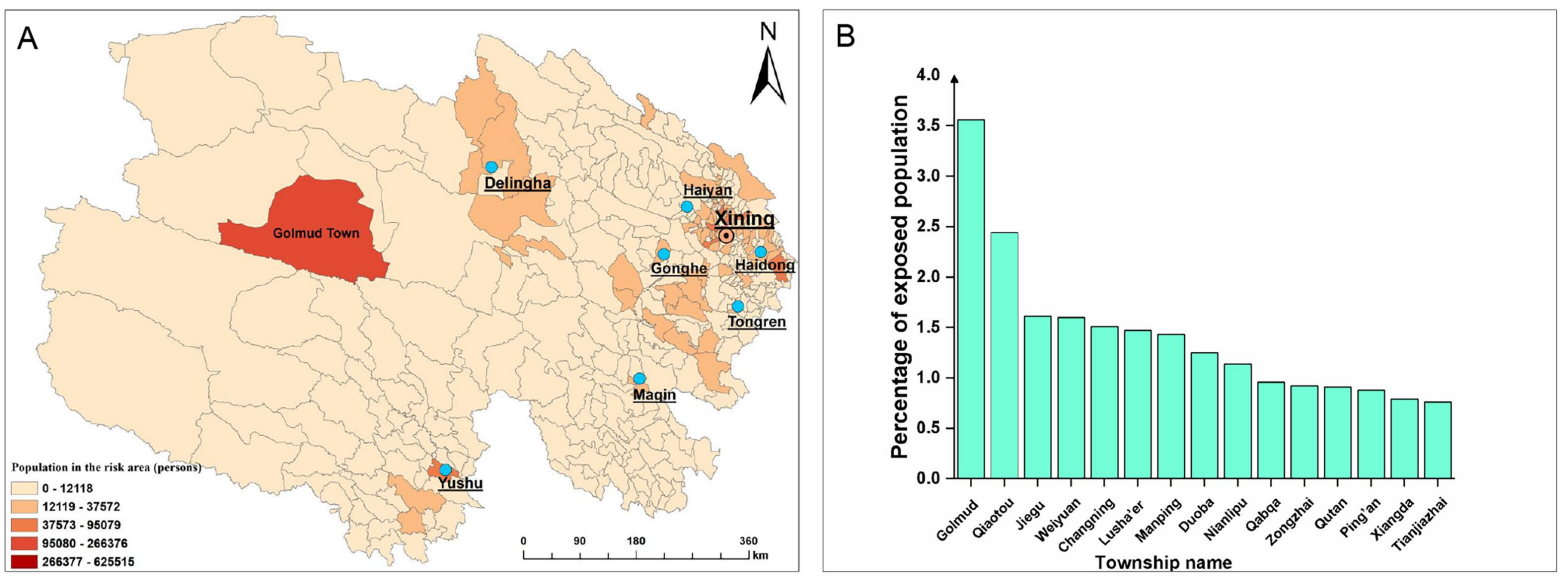
Spatial distribution and proportion of exposed population in townships of Qinghai Province. **(A)**: Spatial distribution of plague risk-exposed population; **(B)**: Top 15 townships by exposed population percentage.

The plague risk zones in Qinghai Province are primarily concentrated around Qinghai Lake in the east and in Yushu Tibetan Autonomous Prefecture in the south, as shown in Figure 7. By calculating the area and exposed population of these risk zones, we determined that the total area of plague risk zones in Qinghai is 234,000 km^2^ (approximately 32.4% of the province’s total area). Among the top 15 townships with the highest proportion of risk area, Suojia Township has the largest risk area, accounting for 3.39% of the total plague risk zone in Qinghai, as illustrated in Figure 8. The exposed population in the plague risk areas of Qinghai Province is 3.902 million, accounting for 65.8% of the province’s total population. It should be noted that this figure represents the spatial overlap between the model-predicted risk areas and the population distribution, rather than indicating actual infection risk or specific disease burden. Among the top 15 townships with the highest proportion of exposed population, Golmud Town has the largest exposed population, representing 3.56% of the total exposed population in Qinghai, as shown in Figure 9.

## Discussion

Existing studies have used ecological niche models (MaxEnt) combined with ecological environmental variables to predict the spatial distribution of Himalayan marmots in localized plague foci within Qinghai Province. However, these studies were mostly confined to small-scale areas and primarily focused on spatial predictions of marmots’ climate-suitable habitats, with relatively few addressing spatial predictions of animal plague risk zones. To address this research gap, this study innovatively integrated 650 unique plague foci in Qinghai Province and their associated ecological environmental variables. By combining principal component analysis (PCA) with the maximum entropy model, we predicted the probability of animal plague occurrence in Qinghai at a 1-km spatial resolution. Based on the 10th percentile logistic training threshold, we classified plague risk and non-risk zones and further quantified the area and exposed population within risk zones. The results showed an average AUC value of 0.827 on the testing datasets and an average TSS of 0.5, indicating the model’s effectiveness in predicting animal plague probability in Qinghai. Spatial analysis revealed that the total area of animal plague risk zones in Qinghai is 234,000 km^2^ (32.4% of the province’s total area), primarily distributed in the eastern and southwestern regions. The exposed population within these risk zones totals 3.902 million people (65.8% of Qinghai’s total population). The spatial distribution map of animal plague risk zones developed in this study, along with supporting statistics on risk area and exposed population, not only enables precise identification of key areas for plague control but also provides high-resolution spatial reference data for early warning systems and targeted prevention strategies for human plague outbreaks.

When multicollinearity exists among continuous environmental variables (e.g., temperature, precipitation, elevation), it can lead to model overfitting. Principal component analysis (PCA) can improve the predictive accuracy of the MaxEnt model in such cases (47). Previous studies typically addressed this issue using variable correlation screening methods. The general workflow involves: (1) assessing collinearity strength among variables via Pearson correlation matrices, (2) identifying highly correlated variable groups based on a predefined threshold (e.g., |r| > 0.7), and (3) retaining representative variables while removing redundant ones based on statistical contributions (31). However, this approach has notable limitations. First, simply discarding variables may result in information loss, particularly for variables with potential ecological significance. Second, the screening process relies heavily on subjective judgment, compromising the stability and reproducibility of model results. In contrast, PCA employs mathematical transformations to convert original variables into uncorrelated principal components. This not only eliminates collinearity among continuous variables but also maximizes the retention of original data information, providing the MaxEnt model with more objective and stable input variables (48). Thus, for spatially predicting animal plague risk zones in Qinghai Province—where diverse geographic environmental variables and human disturbance factors must be integrated—PCA demonstrates clear advantages.

Based on the results of the PCA and MaxEnt models, this study further elucidates that the occurrence of animal plague in Qinghai Province is a complex ecological process jointly driven by specific climatic conditions, topography, and human activities. First, suitable temperatures and topographic features (i.e., warmer winters and mid-to-low altitudes) not only enhance the overwintering survival rate of the primary host, the Himalayan marmot (20), but also provide ideal habitat conditions for the rapid proliferation of vector fleas and the transmission of the pathogen, thereby elevating the risk of animal plague occurrence (49). Second, precipitation patterns play a crucial restrictive role; relatively lower precipitation during the wettest month effectively prevents the destruction of marmot burrow systems (50), while stable precipitation seasonality ensures the continuous growth of alpine meadow vegetation, thereby maintaining a relatively stable host population density, which provides a solid ecological foundation for the persistence of animal plague (51). Furthermore, animal plague exhibits a pronounced spatial tendency to occur in areas closer to human settlements. This suggests that secondary habitat alterations induced by human activities such as grazing (e.g., reduced vegetation height) may lead to the localized aggregation of host populations, consequently increasing the risk of plague exposure for local residents (52). Therefore, clarifying the environmental factors and anthropogenic disturbances affecting the prevalence of animal plague provides a robust scientific basis for developing ecology-based early warning systems and implementing precise, effective animal plague prevention and control strategies in Qinghai Province.

The animal plague risk zones identified in this study cover an area of 234,000 km^2^ in Qinghai Province, accounting for 32.4% of the province’s total area. This result exceeds the area of Qinghai’s natural plague foci (200,000 km^2^, 27.74%). The discrepancy primarily stems from methodological differences. Natural plague foci are defined as areas where *Yersinia pestis* has been detected in wild rodents or their ectoparasites (53), and their total area is derived by aggregating regions meeting these criteria. However, this approach is constrained by actual surveillance coverage, potentially omitting some potential plague risk zones. In contrast, this study employed the MaxEnt model, leveraging known animal plague occurrence data alongside associated environmental and human disturbance variables for prediction. This method not only compensates for limited surveillance but also enables more precise identification of risk zones at the township level. Consequently, the resulting animal plague risk area is more comprehensive and accurate.

The spatial analysis revealed significant regional disparities in the distribution of animal plague risk zones. High-altitude western regions (e.g., Tanggula Town, Suojia Township) and central areas (e.g., Qiejia Township) exhibit extensive risk zones due to their suitable alpine meadow environments, which provide ideal habitats for host animals (54). Notably, a spatial mismatch exists between risk zones and population distribution. While the western and central risk zones cover vast areas, their sparse populations result in fewer exposed individuals. Conversely, in eastern valley regions (e.g., Tangchuan Town), smaller risk zones concentrate larger exposed populations owing to dense settlements and frequent agricultural/pastoral activities (55). This distribution pattern, shaped by both natural geographic conditions and anthropogenic factors, underscores the need for differentiated prevention strategies in Qinghai: Western/Central large-risk zones: Strengthen animal plague surveillance to prevent outbreaks in animal populations. Eastern densely populated areas: Prioritize monitoring of animal-to-human transmission pathways and enhance public health interventions. Such targeted approaches will enable precision in plague control efforts.

The methodology employed in this study demonstrates significant advantages. Firstly, it addresses the limitations of traditional approaches, which require substantial human and material resources and have restricted surveillance coverage—particularly in sparsely populated or remote areas prone to monitoring gaps. Secondly, the 1-km high-resolution risk prediction results overcome the constraints of previous township-level coarse-scale prevention strategies, enabling precise identification of risk distribution patterns within townships. Additionally, based on the spatial distribution characteristics of risk zone areas and exposed populations, this study proposes differentiated prevention strategies: strengthening animal plague surveillance in high-risk zones to enhance early warning mechanisms for human plague outbreaks in high-exposure population areas. This approach optimizes the allocation of prevention and control resources. The high-precision spatial assessment method for animal plague risk zones developed in this study not only improves the targeting and effectiveness of plague control efforts but also provides a scientific foundation for establishing a coordinated “zoonotic surveillance–human outbreak early warning” system.

This study has the following limitations. First, due to vast territory of Qinghai Province, plague surveillance still relies on ground-based manual surveys, making it impossible to achieve comprehensive coverage. Consequently, some animal plague foci may have been overlooked, leading to potentially incomplete training samples for the model. This data limitation could, to some extent, introduce uncertainty into the prediction results. Second, the model assumes that the ecological niche requirements of reservoir hosts remain stable; however, in reality, rodents may alter their distribution patterns through behavioral or genetic adaptations (e.g., pesticide resistance) (56), potentially affecting the accuracy of the identified risk zones. Despite these limitations, this study provides high-precision predictions of risk zones and assesses the resident population within them, thereby offering important scientific evidence and decision support for targeted plague prevention and the optimal allocation of resources in Qinghai Province.

## Conclusion

This study employed the MaxEnt model integrated with GIS spatial analysis to predict animal plague risk zones in Qinghai Province and estimate their area and exposed population. Traditional statistical methods based on administrative divisions, constrained by limited surveillance coverage, fail to comprehensively reflect the spatial distribution of plague risk zones in Qinghai. The approach adopted in this study effectively addresses this gap, providing a scientific basis and decision-making support for targeted plague prevention and control in the region.

## Data Availability

The raw data supporting the conclusions of this article will be made available by the authors, without undue reservation.
