# Supplementary material for: Spatial prediction of animal plague risk in Qinghai Province, China using MaxEnt modeling: implications for targeted control
Source: Front Vet Sci. 2026 Apr 28;13:1737541. doi: 10.3389/fvets.2026.1737541 (PMC13160725; doi:10.3389/fvets.2026.1737541)
Supplement: Supplementary file 1 [file Table_1.DOCX]

**Table S1** Environmental and anthropogenic variables used in the present study.

| Environmental variable | Description | Unit |
| --- | --- | --- |
| Bio1 | Annual mean temperature | ℃ |
| Bio2 | Mean diurnal range | ℃ |
| Bio3 | Isothermality | - |
| Bio4 | Temperature seasonality | - |
| Bio5 | Max temperature of warmest month | ℃ |
| Bio6 | Min Temperature of coldest month | ℃ |
| Bio7 | Temperature annual range | ℃ |
| Bio8 | Mean temperature of wettest quarter | ℃ |
| Bio9 | Mean temperature of driest quarter | ℃ |
| Bio10 | Mean temperature of warmest quarter | ℃ |
| Bio11 | Mean temperature of coldest quarter | ℃ |
| Bio12 | Annual precipitation | mm |
| Bio13 | Precipitation of wettest month | mm |
| Bio14 | Precipitation of driest month | mm |
| Bio15 | Precipitation seasonality | - |
| Bio16 | Precipitation of wettest quarter | mm |
| Bio17 | Precipitation of driest quarter | mm |
| Bio18 | Precipitation of warmest quarter | mm |
| Bio19 | Precipitation of coldest quarter | mm |
| DEM | Altitude | m |
| Aspect | Aspect | ° |
| Slope | Slope | ° |
| D_LST | Daytime land surface temperature | ℃ |
| N_LST | Nighttime land surface temperature | ℃ |
| PopDen_2020 | Population density in 2020 | persons/km² |
| NDVI_2020 | Normalized difference vegetation index in 2020 | - |
| RoadDist | Distance to the nearest road | m |
| RivDist | Distance to the nearest river | m |
| SetDist | Distance to the nearest settlement | m |
| Landform | Geomorphological type | - |
| VegType | Vegetation type | - |
| LandCover | Land cover type | - |
| Soil type | Soil type | - |

**Table S2** Principal component loading matrix of the environmental and anthropogenic variables.

| Layer | PC1 | PC2 | PC3 | PC4 | PC5 |
| --- | --- | --- | --- | --- | --- |
| Aspect | -0.031 | -0.038 | 0.030 | -0.034 | 0.675 |
| Bio1 | 0.679 | -0.415 | -0.110 | 0.005 | -0.016 |
| Bio10 | 0.759 | -0.249 | -0.078 | -0.021 | 0.008 |
| Bio11 | 0.521 | -0.585 | -0.143 | 0.042 | -0.050 |
| Bio12 | -0.663 | -0.423 | 0.038 | 0.001 | -0.054 |
| Bio13 | -0.703 | -0.342 | 0.041 | 0.061 | -0.049 |
| Bio14 | -0.657 | -0.270 | -0.059 | -0.310 | 0.051 |
| Bio15 | -0.515 | 0.218 | -0.162 | 0.487 | 0.030 |
| Bio16 | -0.694 | -0.367 | 0.044 | 0.049 | -0.055 |
| Bio17 | -0.620 | -0.381 | -0.016 | -0.298 | 0.024 |
| Bio18 | -0.694 | -0.366 | 0.044 | 0.049 | -0.055 |
| Bio19 | -0.604 | -0.394 | -0.015 | -0.301 | 0.023 |
| Bio2 | 0.530 | -0.083 | 0.556 | 0.095 | -0.041 |
| Bio3 | -0.218 | -0.479 | 0.407 | 0.249 | -0.174 |
| Bio4 | 0.634 | 0.425 | 0.078 | -0.096 | 0.089 |
| Bio5 | 0.773 | -0.212 | -0.039 | -0.025 | 0.005 |
| Bio6 | 0.483 | -0.515 | -0.337 | 0.027 | -0.060 |
| Bio7 | 0.677 | 0.211 | 0.290 | -0.066 | 0.069 |
| Bio8 | 0.759 | -0.248 | -0.064 | -0.019 | 0.007 |
| Bio9 | 0.584 | -0.503 | -0.059 | -0.047 | -0.072 |
| D_LST | 0.691 | -0.202 | 0.037 | 0.033 | 0.053 |
| DEM | -0.712 | 0.253 | 0.058 | 0.010 | -0.039 |
| NDVI_2020 | -0.313 | -0.479 | 0.106 | 0.058 | 0.077 |
| N_LST | 0.587 | -0.378 | -0.172 | 0.041 | -0.083 |
| PopDen_2020 | 0.019 | -0.031 | -0.067 | 0.093 | -0.041 |
| Slope | -0.300 | -0.253 | -0.004 | -0.060 | 0.073 |
| RoadDist | -0.086 | 0.490 | -0.198 | -0.088 | -0.225 |
| RivDist | 0.367 | 0.072 | 0.343 | -0.332 | -0.208 |
| SetDist | -0.075 | 0.562 | -0.197 | -0.144 | -0.173 |

Note: High component loadings (absolute value > 0.4) are highlighted in red.
